# How to trust size distributions obtained by single particle inductively coupled plasma mass spectrometry analysis

**DOI:** 10.1007/s00216-022-04215-z

**Published:** 2022-07-30

**Authors:** Ana C. Gimenez-Ingalaturre, Khaoula Ben-Jeddou, Josefina Perez-Arantegui, María S. Jimenez, Eduardo Bolea, Francisco Laborda

**Affiliations:** Group of Analytical Spectroscopy and Sensors (GEAS), Institute of Environmental Sciences (IUCA), University of Zaragoza, Pedro Cerbuna 12, 50009 Zaragoza, Spain

**Keywords:** Single particle, SP-ICP-MS, Nanoparticles, Validation, Quality control

## Abstract

Single particle inductively coupled plasma mass spectrometry (SP-ICP-MS) is a technique widely used to obtain direct information about the number concentration and the size distribution of nanoparticles in liquid suspensions. However, its methods still lack clear quality control strategies to confirm the validity of the information derived from them. Only the detection of the complete size distribution of the nanoparticles in a sample over the size critical value ensures obtaining unbiased quantitative information, otherwise information should be restricted to report the presence of nanoparticles over a certain size and number concentration since their actual total number concentration is underestimated and the size overestimated. Under the latter conditions, data processing produces histograms showing the tails of the incomplete size distributions, although apparently, complete distributions can also be obtained when particle events are recorded as peaks, as reported here for the first time. The occurrence of these misleading situations must be critically evaluated for each SP-ICP-MS analysis. An approach, based on estimation of size critical values and successive dilutions, is proposed for the assessment of the validity of the quantitative information obtained, together with specific criteria for reconsidering the information that can be derived from those measurements. The approach was verified with different case studies and applied to the analysis of complex nanomaterials, confirming the validity of the reported information by comparison with other techniques. A calculation tool is also included to facilitate the estimation of size critical values under experimental conditions.

**Graphical abstract:**

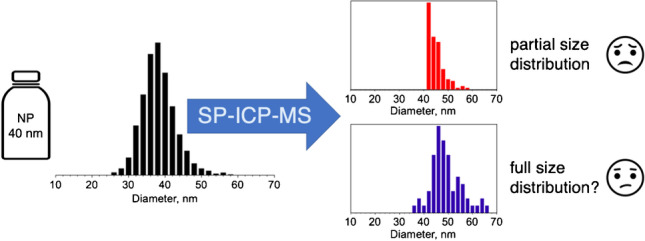

**Supplementary Information:**

The online version contains supplementary material available at 10.1007/s00216-022-04215-z.

## Introduction

Single particle detection has become one of the cornerstones of inductively coupled plasma mass spectrometry (ICP-MS) over the last years, offering unique features for the analysis of (nano)particle suspensions [1]. Whereas conventional ICP-MS just provides information about elemental composition and element mass concentration, an ICP-MS instrument working in single particle mode allows to obtain: (i) qualitative information about the presence of (nano)particulate and dissolved forms of specific elements; (ii) characterization information about the mass of element/s per (nano)particle, which can be converted into particle size as long as information about the composition, shape, and density of the (nano)particles is known or assumed; and (iii) quantitative information as number concentration of (nano)particles, as well as mass concentrations of the dissolved and (nano)particulate forms [2]. The additional work of manufacturers, by incorporating technical improvements to the ICP-MS instruments, as well as specific software for the treatment of data, has contributed to the spreading of single particle ICP-MS (SP-ICP-MS) [3–5].

In any case, the development of SP-ICP-MS during the last years has made this methodology available to a broad range of users and applications. SP-ICP-MS applications can be divided into two main groups: (i) laboratory studies where the fate and transformations of nanoparticles spiked to in vitro and in vivo assays are followed in test media or organisms along the assays (e.g., environmental, ecotoxicological, and toxicological studies); and (ii) analysis of industrial and consumer products containing nanoparticles (e.g., cosmetics, textiles, polymers, and foods) and monitoring the occurrence of (nano)particles in the environment and organisms, including humans. Whereas in the first group of applications the chemical composition, concentration, and size of the (nano)particles are well known, this is not the case when analyzing any of the samples from the second group. This means that users need basic and robust criteria and tools to confirm the information provided by SP-ICP-MS methods in such analysis. Because of the inherent difficulties when analyzing such unknown samples, SP-ICP-MS users must be aware of the current limitations of this technique and take one step backward, focusing primarily on the level of information obtained and the extent to which a SP-ICP-MS method applied to a specific sample provides the information what is intended to be reported.

Considering one specific element, the first question should be: Are there particles containing such element in the sample? If the answer is affirmative, the next questions could be: What is their mass content per particle (or their size if the shape, composition, and density of the particles is known or assumed)? and what is the concentration of particles? The first question can be answered by using SP-ICP-MS as screening method and applying a number of metrological criteria [6]. Answering the next questions will depend on the actual population of particles in the sample and the capability of the instrument to record their full-size distribution in such sample.

If the particles in the sample are larger than the size critical value, accurate information about the complete size distribution and the number concentration of the particles may be obtained, otherwise just partial information will be attainable, being restricted to particles over the size critical value and hence underestimating their actual total number concentration. This information could also be considered acceptable if the user is aware of its limitations. Whereas in scenario (i) SP-ICP-MS results can be compared with data from the spiked nanoparticles [7–10], this is not the case in scenario (ii), where the use of alternative and/or supplementary methods are needed for confirmation of the SP-ICP-MS results [11–13].

Apart from their concentration, the detection of particles in SP-ICP-MS is conditioned by the element measured and its content in the particle, as well as the performance of the instrument (detection efficiency) [14, 15]. On the other hand, particle events in SP-ICP-MS are detected as pulses or peaks over a continuous baseline, whose origin is the instrumental background or the presence of dissolved forms of the element measured. In any case, the noise associated to the baseline constrains the capability of detection of particles, but it can also lead to the occurrence of false positives when threshold and peak detection criteria are not applied conveniently. Whereas particle events detected as pulses (one-reading events) by using dwell times in the range of milliseconds are easily handled by using simple algorithms implemented in spreadsheets, data recorded at faster frequencies using microsecond dwell times involve processing of the peaks (more than one-reading events) by more complex algorithms and software, which should also be validated, in line with those used in chromatography [16].

Although most of the application of SP-ICP-MS described in the bibliography use the commercial software provided by the instrument companies for the detection of particle events, different alternatives have been proposed working with dwell times both in milli- [17] and microseconds [17–21]. Most of these approaches try to improve the identification of nanoparticles with sizes close to the limit of detection or in those situations with high concentrations of dissolved forms of the element measured. Basically, both Gaussian [18, 19, 21] and Poisson [20, 21, 4

The aim of this work is to assess critically the information achieved by SP-ICP-MS and how it should be reported to maintain its validity. Although main attention is paid to the effect of the baseline on the capability of detection of particles and the misinterpretation of the signals obtained, the study is not limited to those situations with high dissolved element concentrations, but give an overall quality control strategy based on successive dilutions in combination with the estimation of size critical values. This strategy proposed has been checked for assessing the validity of the reported SP-ICP-MS information in complex nanomaterials, by comparison with other techniques.

## Experimental

### Instrumentation

A Perkin-Elmer NexION 2000 mass spectrometer (Toronto, Canada) was used for ICP-MS measurements in single particle mode. The sample introduction system consisted of a glass concentric nebulizer and a baffled cyclonic spray chamber (Meinhard). Default instrumental and data acquisition parameters are listed in Table 1.

**Table 1:** Default instrumental and data acquisition parameters for SP-ICP-MS

Instrumental parameters	
RF power	1600 W
Argon gas flow rate	
Plasma	15 L min^−1^
Auxiliary	1.2 L min^−1^
Nebulizer	1.0 L min^−1^
Sample flow rate	0.34 mL min^−1^
Data acquisition parameters	
Dwell time	100 µs, 5 ms
Total acquisition time	60, 300 s
Isotopes monitored	^107^Ag, ^197^Au

Separations by hydrodynamic chromatography (HDC) were performed on a Waters 2796 chromatograph (Bioseparation module, Waters Corporation, Milford, USA) coupled to an ICP-MS ELAN DRC-e (Perkin-Elmer, Toronto, Canada). The chromatograph was equipped with a non-porous packed column PL-PSDA type 1 (Agilent Technologies, Germany) for hydrodynamic separations, with a nominal separation range from 5 to 300 nm. The column dimensions were 80 cm in length and 7.5 mm of internal diameter.

A field-emission scanning electron microscope (Merlin™ FESEM, Carl Zeiss Nano Technology Systems, Jena, Germany) with a Gemini column and an energy-dispersive X-ray microanalyzer (X-Max, Oxford Instruments, Abingdon, UK) was used to imaging the samples. Observations were carried out at 5 kV. A FEI Tecnai T20 Transmission Electron Microscope (FEI Technologies Inc., USA), working at 200 kV, was also used.

### Standards

Diluted suspensions of gold and silver nanoparticles were prepared from commercially available suspensions. An ultra-uniform gold nanoparticle (PEG-carboxil 0.8 kDa surface) suspension of 47.8 ± 1.8 nm diameter was obtained from NanoComposix (San Diego, CA, USA). Suspensions of monodisperse citrate-stabilized silver nanoparticles of nominal diameter 10.3 ± 2.1, 20.8 ± 3.0, 39 ± 5, and 60 ± 7 nm were purchased from NanoComposix (San Diego, CA, USA). Sodium dodecylsulphate (SDS) (Bio-Rad, California, USA), sodium hydroxide (Scharlau, Barcelona, Spain), D-penicillamine (Sigma Aldrich, Germany), and nitric acid (Baker Instranalyzed for Trace Metals Analysis, J.T. Baker, Holland) were also used.

Aqueous gold and silver solutions were prepared from standard stock solutions of 1000 mg L^−1^ (Sigma Aldrich, Switzerland) by dilution in ultrapure water.

### Nanomaterials

Two antimicrobial products, denoted as M1 and M2, consisting of aqueous suspensions containing silver nanoparticles stabilized with a natural organic ligand, were provided by Laboratorios Enosan S.L., Spain.

### Procedures

#### Standard suspensions

Dilutions of the stock suspension of silver and gold nanoparticles were prepared in ultrapure water (Milli-Q Advantage, Molsheim, France) by accurately weighing (± 0.1 mg) aliquots after 1 min sonication. After dilution and before each analysis, the suspensions were bath sonicated for 1 min. Aliquots of silver (I) solution were added in concentrations from 0.02 up to 2.50 μg L^−1^ to silver nanoparticles suspensions, which number concentration was kept constant in each experiment. Samples were bath sonicated for 1 min before each analysis.

#### SP-ICP-MS measurements

Suspensions were measured in single particle mode using the Syngistix Nano-Application module version 2.5 (PerkinElmer, Inc.). The dwell times used were 5 ms and 100 μs with total acquisition times of 60 and 300 s, recording 12 000 and 60 000 (at 5 ms) or 600 000 and 3 million (at 100 μs) readings per time scan, respectively (Table 1). Nebulization efficiency was determined using the ultra-uniform gold nanoparticle standard described above. Similar results were obtained for the frequency and the size methods. Sample flow rate was measured gravimetrically. Recorded signals were initially processed by using the software provided by the manufacturer (Syngistix Nano-Application module version 2.5) by applying a 5-sigma threshold calculated as five times the square root of the mean baseline intensity of the time scan. Alternatively, recorded scan files were exported and processed with the SPCal software [21] by using the Poisson filter option.

#### Analysis by HDC-ICP-MS

Volumes of 50 μL of the antimicrobial nanomaterials, diluted to ca. 300 µg L^−1^ with 1 mM penicillamine, were directly injected in the HDC column. The mobile phase consisted of 0.34 mM sodium dodecyl sulphate and 1 mM penicillamine [23]. The mobile phase was previously filtered through a 0.22 µm filter and degassed through an online vacuum degasser. Table S1 of Supplementary Information summarizes the experimental conditions.

For chromatograms integration and data processing, Origin 8 was used (Origin Lab, Northampton, MA, USA).

#### Size characterization by FESEM and TEM

A volume of 20 µL of sample was deposited on a copper-grid holder, dried at room temperature, and carbon-coated using a Leica EM SCD500 high vacuum sputter coater (Leica Microsystem, Vienna, Austria) to improve conductivity. The same preparations were used for FESEM and TEM observations. ImageJ (Version 1.52) software was used for image processing and nanoparticle diameter measurement.

### Calculation of size critical values

Size critical values (${X}_{C}^{size}$) were estimated according to the 5-sigma threshold criterion used for discrimination of baseline from nanoparticle events, using different expressions depending on the dwell time applied and the type of particle event recorded [14]. For particle events recorded as pulses by using millisecond dwell times, the following expression was used:

1 $$\mathrm X_{\mathrm C}^{\mathrm{size}}=\left(\frac{{30\sigma}_{\mathrm B}}{\pi\rho{\mathrm F}_{\mathrm P}{\mathrm K}_{\mathrm{ICPMS}}{\mathrm K}_{\mathrm M}}\right)^{1/3}$$

where ${\sigma }_{B}$ is the standard deviation of the baseline, $\rho$ is the density, ${F}_{P}$ is the mass fraction of the element in the particle, ${K}_{ICPMS}$ is the detection efficiency, which represents the ratio of the number of ions detected versus the number of analyte atoms of the measured isotope introduced into the ICP; and ${K}_{M}$ *(*${=AN}_{Av}/{M}_{M}$*)* is a factor related to the element measured, where $A$ is the atomic abundance of the isotope considered, ${N}_{Av}$ is the Avogadro number, and ${M}_{M}$ is the atomic mass of the element*.* For particle events recorded as peaks by using microsecond dwell times, the expression was:

2 $$\mathrm X_{\mathrm C}^{\mathrm{size}}=\left(\frac{{30\sigma}_{\mathrm B}}{\frac2{\mathrm w}{\pi\rho{\mathrm F}_{\mathrm P}\mathrm K}_{\mathrm{ICPMS}}{\mathrm K}_{\mathrm M}{\mathrm t}_{\mathrm{dwell}}}\right)^{1/3}$$

where $w$ is the time-width of the peak (ca. 500 µs under the conditions used) and ${t}_{dwell}$ is the dwell time. In both cases, ${\sigma }_{B}$ was calculated as the square root of the mean baseline intensity (${Y}_{B}$), measured in counts (per dwell time), rounded up to the next integer to be in accordance with the threshold criterion applied for discrimination of particle and baseline readings [14]. ${K}_{ICPMS}$ was calculated from the analytical sensitivity of the dissolved element, the analyte nebulization efficiency, and sample flow rate, following a procedure described elsewhere [24] by using a dedicated spreadsheet. This spreadsheet (SP-ICP-MS_LODs_2.2) is available as Supplementary Information, and it is a revised version of SP-ICP-MS_LODs_1.3 [14], in which size critical values and limits of detection were calculated from the upper integer of the square root of the mean baseline intensity instead of just from its square root. Both size critical values and limits of detection were calculated from the same expression, which involves a 50% probability of false negatives in the calculation of the limits of detection, an assumption justified elsewhere [14, 25]. In the context of this work, it should be noted that the value calculated from Eq. 2 must be quoted as an a posteriori critical value or limit of decision and not as an a priori limit of detection, since the expression is going to be used to make the decision whether nanoparticles above a certain size have been detected in a sample.

## Results and discussion

### Effect of size critical values on the recording of nanoparticle size distributions

Nanoparticles occur as more or less broad size distributions. This means that information about the content of nanoparticles in a sample is conditioned not only by the capability of the SP-ICP-MS method to detect low concentrations of nanoparticles but also by its capability of detecting the complete distribution, including the smallest nanoparticles of it. On the other hand, size critical values and limits of detection in SP-ICP-MS depend not only on the performance of the instrument used and the element monitored but also on the baseline through its associated noise [14].

**Fig. 1:**
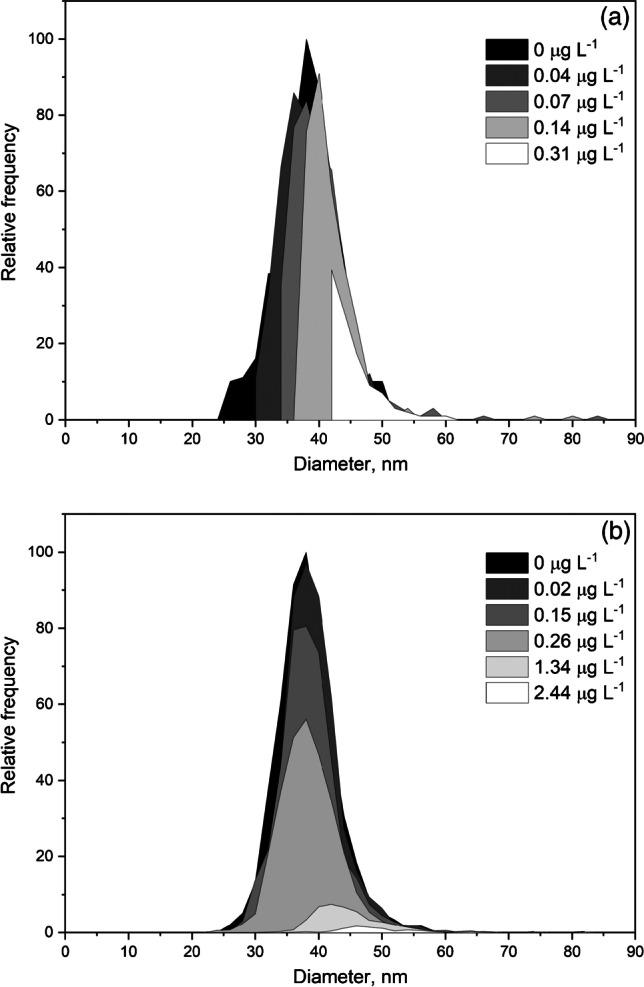
Size distributions of 40 nm silver nanoparticles spiked with increasing concentrations of ionic silver for dwell times of 5 ms (**a**) and 100 µs (**b**). The legends list the ionic silver concentration in the nanoparticle suspensions

The effect of the baseline on the distribution recorded can be seen in Fig. 1. This figure shows the size distributions of 40 nm silver nanoparticles obtained at different mean baseline intensities by increasing the concentration of ionic silver from 0.02 to 2.50 µg L^−1^, affecting the size critical value. Nanoparticle events were detected over the continuous baseline by using the software of the manufacturer applying a threshold criterion of 5-sigma, where the threshold was calculated from the mean baseline intensity measured in counts (${Y}_{B}$) as ${Y}_{B}+5\sqrt{{Y}_{B}}$ (rounded up to the upper integer), because of the Poisson behavior of the baseline [6]. Size critical values were calculated according to the same threshold criterion, applying Eqs. 1 and 2 for particle events recorded as pulses or peaks, respectively.

When a dwell time of 5 ms was used (Fig. 1a), the distribution was progressively lost when the baseline intensity increased. In this case, the size critical value increased from 23 nm, for a baseline corresponding to ultrapure water, up to 41 nm due to the presence of dissolved silver. When compared to the original size distribution of the nanoparticles (in black), the distribution was partially recorded because signals from nanoparticles below 41 nm could not be discriminated from the baseline noise. In spite of this, the appearance of the recorded distribution confirmed at first sight that part of the nanoparticle distribution had been missed and quantitative information about the nanoparticles in the suspension was not feasible. When the same nanoparticles were measured at 100 µs dwell time (Fig. 1b), the results were histograms showing apparently complete size distributions, although their magnitude decreased and their maximum moved to larger sizes when the concentration of dissolved silver and hence the intensity of the baseline increased. In the absence of the original size distribution of the suspension in ultrapure water, and no further confirmation of the results obtained, the concentration of nanoparticles in some of these suspensions would be underestimated and their mean size overestimated. When comparing Figs. 1a and b, the main difference is that the distributions obtained in Fig. 1a are self-explanatory, showing that the distributions have been partially recorded, whereas those from Fig. 1b are not.

This different appearance of the size distributions when they are measured at milli- and microsecond dwell times lies in the nature of the particle events, pulses, or peaks, and how they have been processed. By using the manufacturer software, the application of any threshold criterion to pulse events directly removes those below the threshold, whereas when working with peak events, they are “partially lost” in the baseline noise, and their net total intensity reduced. The result is that large peaks are going to become smaller and small peaks are going to be missed, moving the size distribution to larger sizes, detecting less particles, but maintaining an apparent complete size distribution. Similar results were obtained when the raw data were processed by using the open-source software SPCal [21], as shown in Fig. S1.

Table 2 summarizes the effect of increasing the baseline intensity on the determination of the mean size of the nanoparticles and their number concentration by using the manufacturer software. The results obtained by using the software SPCal are presented in Table S2. With both data treatments, the increase in the size critical value involved an apparent increase in the mean size and a reduction in the number concentration, which was directly related to the progressive loss of the smaller nanoparticles. This means that, when analyzing unknown samples by SP-ICP-MS, the results obtained must be assessed in some way to confirm their validity. Otherwise, the information obtained should be reported just as qualitative or semiquantitative, and only the presence of particles over a certain size or/and number of particles over a certain number concentration [6] could be confirmed. Whenever the size distributions are not fully recorded, this should always be the rule.

**Table 2:** Mean size and number concentration of nanoparticles, size critical values (${X}_{C}^{size}$), and nanoparticle recovery for 40 nm silver nanoparticles spiked with increasing concentrations of ionic silver for dwell times of 5 ms and 100 µs. Total acquisition time: 60 s. Mean ± standard deviation (*n* = 3)

Suspension	Mean baseline intensity counts	$\mathrm X_{\mathrm C}^{\mathrm{size}}$ nm	Mean size nm	Number concentration L^−1^	Recovery %
Dwell time: 5 ms					
40 nm AgNPs	2	22.9	39.1 ± 0.2	1.64 × 10^7^ ± 0.07 × 10^7^	100 ± 4
40 nm AgNPs + 0.04 µg L^−1^ Ag(I)	8	28.8	41.1 ± 0.5	9.8 × 10^6^ ± 0.5 × 10^6^	63 ± 3
40 nm AgNPs + 0.07 µg L^−1^ Ag(I)	13	31.9	44.3 ± 0.6	4.2 × 10^6^ ± 0.2 × 10^6^	27 ± 1
40 nm AgNPs + 0.14 µg L^−1^ Ag(I)	27	35.5	49.3 ± 0.9	1.7 × 10^6^ ± 0.1 × 10^6^	10.3 ± 0.4
40 nm AgNPs + 0.31 µg L^−1^ Ag(I)	64	40.9	58.8 ± 0.8	2.4 × 10^5^ ± 1.2 × 10^5^	1.4 ± 0.7
Dwell time: 100 µs					
40 nm AgNPs	0.05	19.4	38.4 ± 0.2	1.50 × 10^8^ ± 0.03 × 10^8^	100 ± 2
40 nm AgNPs + 0.02 µg L^−1^ Ag(I)	0.1	19.4	38.1 ± 0.2	1.37 × 10^8^ ± 0.02 × 10^8^	96 ± 2
40 nm AgNPs + 0.15 µg L^−1^ Ag(I)	0.5	24.4	37.5 ± 0.3	1.27 × 10^8^ ± 0.04 × 10^8^	87 ± 3
40 nm AgNPs + 0.26 µg L^−1^ Ag(I)	1	28.0	38.5 ± 0.1	8.6 × 10^7^ ± 0.2 × 10^7^	59 ± 2
40 nm AgNPs + 1.34 µg L^−1^ Ag(I)	5	35.2	44.1 ± 0.6	1.2 × 10^7^ ± 0.1 × 10^7^	8.4 ± 0.7
40 nm AgNPs + 2.44 µg L^−1^ Ag(I)	11	39.6	51.4 ± 2.4	2.9 × 10^6^ ± 0.1 × 10^6^	1.9 ± 0.1

Although the cases presented involve high intensity baselines due to the presence of dissolved species, the situations would also apply to isotopes with high background levels due to occurrence of polyatomics or even to low intensity baselines if the size of the nanoparticles measured is close to the size detection limit.

### Approach for the assessment of the information provided by SP-ICP-MS analysis

In order to avoid the misinterpretation problems discussed above when unknown samples are analyzed by SP-ICP-MS, the results obtained should be assessed in some way, especially when using microsecond dwell times. Schwertfeger et al. [26] proposed a strategy involving the analysis of samples at different dilutions with the aim of reducing the concentration of dissolved element, despite the total number of particles counted were lower, which was compensated by increasing the acquisition time. Under such conditions, the information obtained would be considered valid if the number concentrations determined at two different dilutions were similar. Aznar et al. [27] also proposed an approach for quality control of the results based on the progressive dilution of the samples while maintaining the size distribution and the particle counts proportional to the dilution applied.

Whereas the approach of Aznar et al. was empirical, checking different dilutions to find the optimal measurement range for each sample, Schwertfeger et al. proposed the dilution of the samples to reduce the dissolved element concentration below the detection limit achieved by standard ICP-MS analysis, in combination with the increase of the acquisition time if needed. However, this criterion should be considered misleading since limits of detection of dissolved elements measured by ICP-MS in single particle mode are much lower than in standard mode, because of the longer acquisition times used in SP-ICP-MS, and the fact that the increase of the acquisition time contributes to reduce this limit of detection further [14]. Our proposal is that, as a rule of thumb, samples should be diluted as much as possible to reduce the baseline intensity to blank levels, in order to get the best available size critical value, or, at least, to values corresponding to size critical values below the lower end of the nanoparticle distribution. Even under conditions of baseline blank levels, if the measured size distribution reaches a value that is next to the size critical value, part of the actual size distribution is being missed and the quantitative information will be biased.

**Fig. 2:**
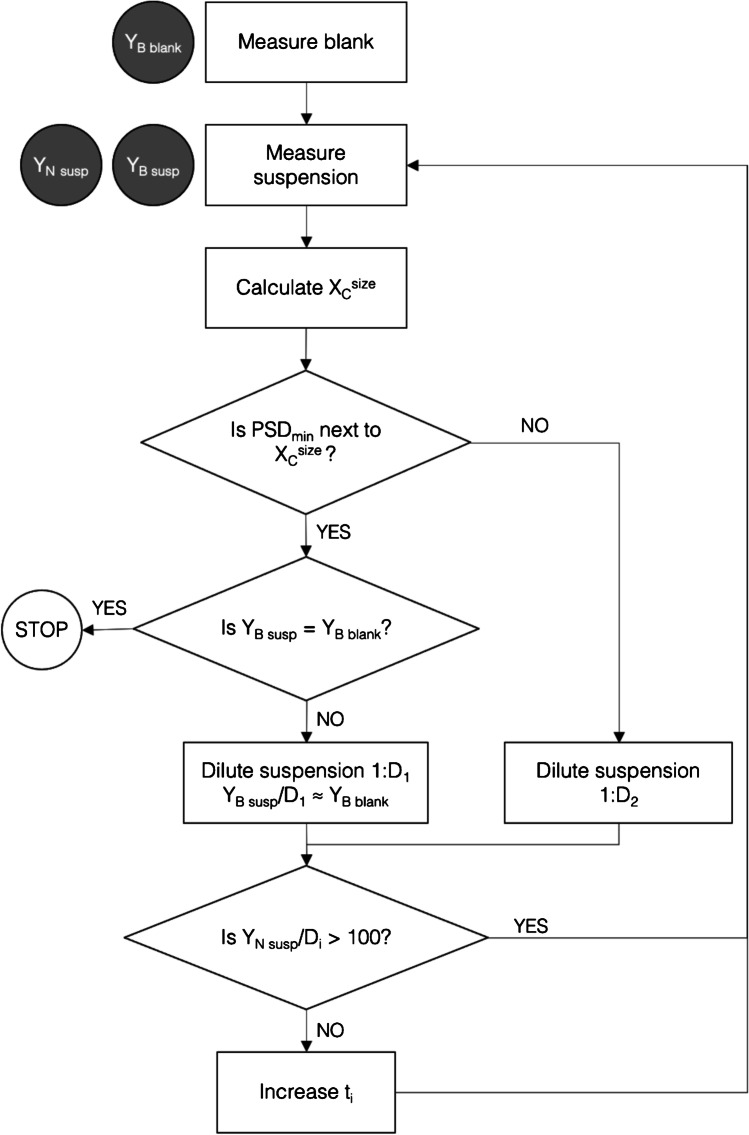
Flow chart for the trueness assessment of the information provided by SP-ICP-MS. ${Y}_{B blank}$: mean baseline intensity of the blank. ${Y}_{B susp}$: mean baseline intensity of the suspension. ${Y}_{N susp}$: number of particle events of the suspension. ${X}_{C}^{size}$: size critical values. ${t}_{i}$: total acquisition time. D_1_ and D_2_: dilution factors. PSD_min_: minimum size of the particle size distribution

Figure 2 shows a flow chart that summarizes the assessment approach proposed. First, the measurement of an instrumental blank (e.g., ultrapure water) allows to obtain the mean baseline intensity of the blank ($${Y}_{B blank}$$) to estimate the best size critical value from Eq. 1 ($${\sigma }_{B}=\sqrt{{Y}_{B blank}}$$). For simplicity, it is considered that no particles are detected from the blank, so the counting of particle events in a sample can be assimilated to an ideal Poisson counting process with zero blank. Under such ideal conditions, the detection of one particle event in a sample time scan would confirm the presence of particles in the sample [28]. Next, by measuring the nanoparticle suspension, information about the mean baseline intensity of the suspension ($${Y}_{B susp}$$) and the number of particle events ($${Y}_{N susp(1)}$$) is obtained. The attainable size critical value in the suspension can be estimated from Eq. 1 or 2 and the measured $${Y}_{B susp}$$. Whenever the lower end of the measured size distribution, defined as the minimum size of the particle size distribution (PSD_min_) with a number of particles higher than 0 (critical value for a zero blank), is larger than the size critical value ($${X}_{C}^{size})$$, the suspension can be diluted (e.g., 1:2) to make a second confirmatory measurement $${Y}_{N susp(2)}$$. On the contrary, if the PSD_min_ is next to the size critical value (the immediately consecutive data), the suspension must be diluted to reduce the baseline to the blank level, unless the baseline level in the blank and the suspension were not statistically different, in which case size critical values cannot be improved by dilution and hence no additional action can be taken. If particles are detected from a blank, a critical value for detection of particles higher than zero should be considered and more complex expressions should be used for its calculation [6, 28].

When measuring diluted suspensions, the acquisition time should be increased conveniently to count at least 100 particle events (minimum number concentration limit of quantitation under ideal counting conditions [28]). The agreement between the number concentrations determined from both dilutions will confirm the information obtained, both as number concentration and size distribution, otherwise part of the original size distribution of nanoparticles is not detected and the information will have to be considered as semiquantitative or just qualitative. In the case of particles with broad distributions, dilution might lead to a loss of information about the distribution itself, since fewer of the largest and the smallest particles would be detected despite increasing the acquisition time.

### Proofs-of-concept of the approach

Two generic cases were studied as proofs-of-concept of the approach presented above. The first one considers the presence of dissolved species, whose effect can be eliminated by dilution, whereas the second one corresponds to a situation where the baseline level in the sample is similar to that in a blank.

**Fig. 3:**
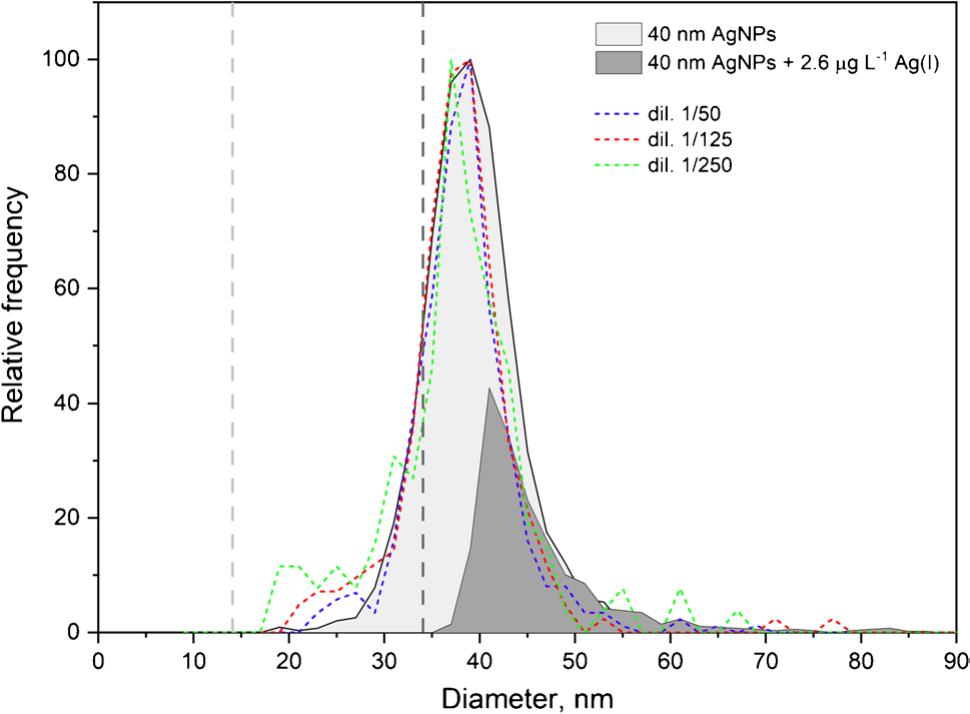
Size distributions of 40 nm silver nanoparticle suspensions, unspiked (light gray) and spiked with 2.6 µg L^−1^ of ionic silver (dark grey), and dilutions of this latter sample. Grey dashed lines: size critical values. Dwell time: 100 µs

With respect to the first case, Table 3 summarizes the steps followed for the assessment of the information obtained from a suspension containing 40 nm silver nanoparticles and 2.6 µg L^−1^ of ionic silver. Critical values reported in Table 3 cannot be compared to those in Table 2 because they were obtained under different sensitivity conditions. Due to the presence of dissolved silver, the actual size critical value in this suspension was 34 nm, which was higher than the lower end of the original size distribution shown in Fig. 1 (ca. 25 nm), and hence the nanoparticles detected accounted for just 15% of the actual distribution. Therefore, a 1:50 dilution was applied to reduce the baseline intensity and the size critical value, although the acquisition time had to be increased from 60 to 300 s to increase the number of events recorded over 100. Under such conditions, the size critical value was reduced to 18 nm, increasing the nanoparticle recovery to 80%. A 1:125 dilution allowed to reduce the baseline intensity close to blank levels (0.2 vs. 0.1 mean counts of baseline), corresponding to a size critical value of 16 nm, which was clearly smaller than the lower end of the distribution (ca. 25 nm), so the complete size distribution could be obtained with quantitative recovery of the nanoparticles. To confirm these results, the last dilution was further diluted 1:2 (up to 1:250 from the original suspension), showing good agreement between both. With respect to the size distributions obtained, Fig. 3 shows that the information from the original suspension was clearly biased, reporting an apparently complete distribution of silver nanoparticles from 35 nm, which only corresponded to the tail of the actual distribution. The successive dilutions showed a fair agreement between them, although the low number of events recorded for the highest dilutions involves a lower precision due to counting statistics.

**Table 3:** Mean size and number concentration of nanoparticles, size critical values (${X}_{C}^{size}$), and nanoparticle recovery for 40 nm silver nanoparticle suspension spiked with 2.6 µg L^−1^ of ionic silver and analyzed at different dilutions and total acquisition times. Dwell time: 100 µs. Mean ± standard deviation (*n* = 3)

Suspension	Acquisition time s	Mean baseline intensity counts	$\mathrm X_{\mathrm C}^{\mathrm{size}}$ nm	Mean size nm	Number of events	Number concentration L^−1^	Recovery %
40 nm AgNPs	60	0.1	14.4	38.0 ± 0.2	3085 ± 26	1.45 × 10^8^ ± 0.01 × 10^8^	100 ± 2
40 nm AgNPs + 2.6 µg L^−1^ Ag(I)	60	27	34.2	46.1 ± 0.3	968 ± 40	4.56 × 10^7^ ± 0.02 × 10^8^	15 ± 1
1:50 dilution	300	0.4	18.1	38.0 ± 0.3	511 ± 16	1.92 × 10^8^ ± 0.06 × 10^8^	80 ± 3
1:125 dilution	300	0.2	16.4	37.6 ± 0.2	244 ± 10	2.7 × 10^8^ ± 0.1 × 10^8^	98 ± 4
1:250 dilution	300	0.1	14.4	36.7 ± 1.0	136 ± 10	3.1 × 10^8^ ± 0.2 × 10^8^	103 ± 4

**Fig. 4:**
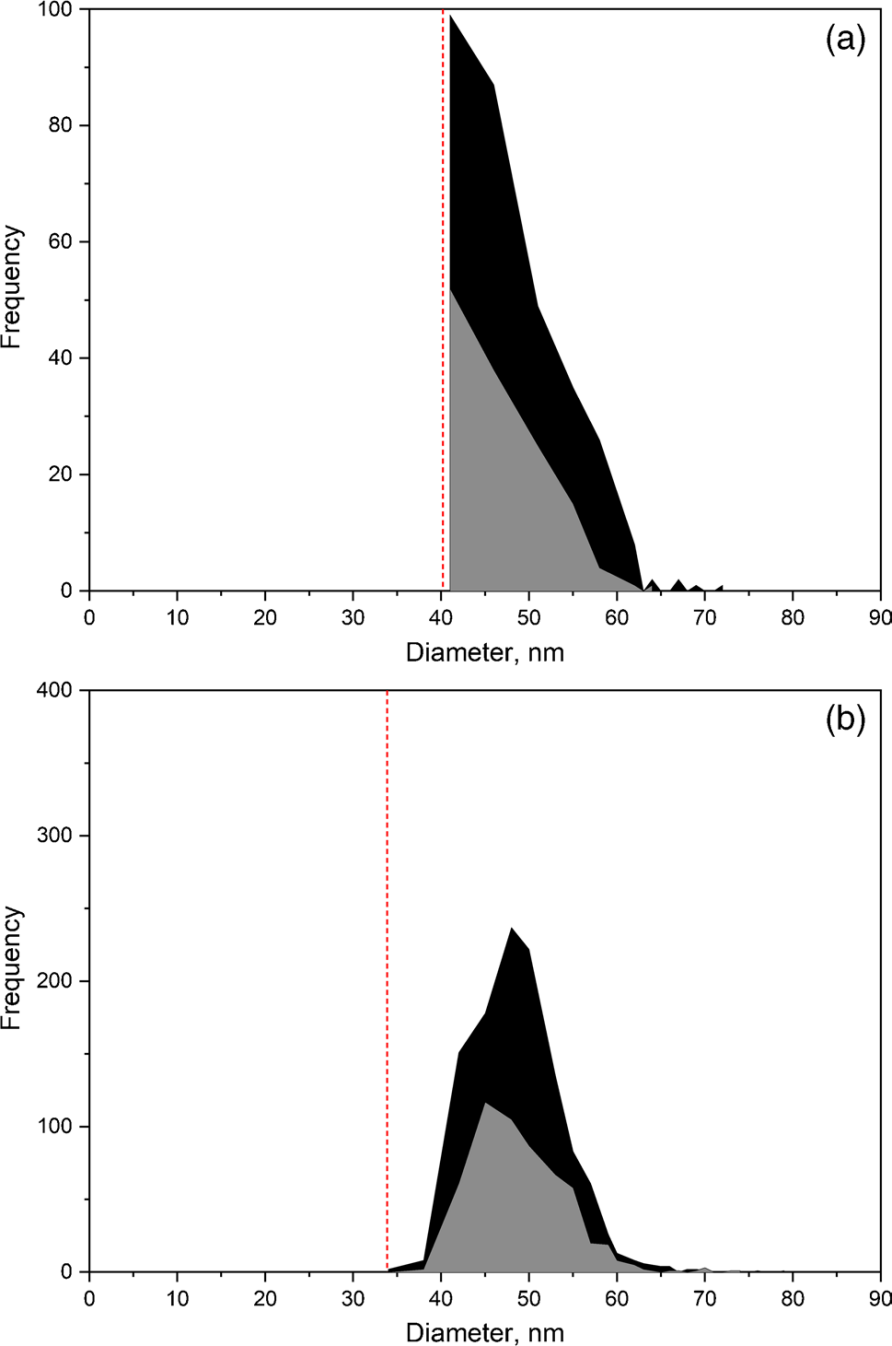
Size distributions of a 50 nm gold nanoparticle suspension (black area) and a 1:2 dilution (grey area) under low sensitivity conditions at (**a**) 5 ms and (**b**) 100 µs dwell times. Red dashed line: size critical value

In the case discussed above, the main contribution to the baseline was due to the dissolved element and dilution allowed to improve size critical values as a consequence, detecting the whole nanoparticle distribution over such critical value. However, when the baseline intensity is negligible (or constant due to plasma polyatomic interferences), size critical values cannot be further improved by dilution. Table 4 shows the effect of dilution on suspensions of 50 nm gold nanoparticles measured at 5 ms and 100 µs dwell times under conditions that provided size critical values lower than the PSD_min_ (11 nm in both cases) or much higher (42 and 34 nm, respectively). This was accomplished by modifying the deflector voltage of the focusing quadrupole of the ICP-MS to reduce the transmission of ions to the mass spectrometer and hence the overall sensitivity. Under such conditions, the recovery of all the dilutions was quantitative, in spite of the nanoparticle distribution recorded under low sensitivity conditions accounted for 78–80% and 63% of the original one at 5 ms and 100 µs, respectively. Whereas the low recoveries achieved at 5 ms were evident from the profile of the distributions recorded (Fig. 4a), this was not the case when working at 100 µs (Fig. 4b) as expected from the behavior presented in the previous section. The direct consequence of these results is that confirmatory measurements based on the dilution of a suspension are no longer valid when the baseline level in the suspension measured is close to that in a blank and the lower limit of the size distribution is similar to the size critical value. Under such conditions, the flow chart in Fig. 2 leads to a STOP and implies that results obtained cannot be confirmed and most probably the suspension contains nanoparticles below the size critical value that have not been recorded. Under such conditions, results must be considered only qualitative or semiquantitative.

**Table 4:** Mean size and number concentration of nanoparticles, size critical values (${X}_{C}^{size}$), and nanoparticle recovery for 50 nm gold nanoparticles at different sensitivities and dwell times of 5 ms and 100 µs. Total acquisition time: 60 s. Mean ± standard deviation (*n* = 3)

Suspension	Mean baseline intensity counts	$\mathrm X_{\mathrm C}^{\mathrm{size}}$ nm	Mean size nm	Number of events	Number concentration L^−1^	Overall recovery %	Dilution recovery %
Dwell time: 5 ms							
50 nm AuNPs	0.1	10.6	48.5 ± 0.4	380 ± 17	1.90 × 10^7^ ± 0.08 × 10^7^		
1:2 dilution	0.1	10.6	47.1 ± 0.7	187 ± 9	1.89 × 10^7^ ± 0.09 × 10^7^		100 ± 5
50 nm AuNPs (low sensitivity)^a^	0.0	41.6	50.4 ± 0.5	296 ± 20	1.48 × 10^7^ ± 0.10 × 10^7^	78 ± 5	
1:2 dilution	0.0	41.6	49.2 ± 0.6	149 ± 7	1.51 × 10^7^ ± 0.07 × 10^7^	80 ± 4	102 ± 5
Dwell time: 100 µs							
50 nm AuNPs	0.0	10.9	48.3 ± 0.3	1770 ± 24	8.68 × 10^7^ ± 0.12 × 10^7^		
1:2 dilution	0.0	10.9	47.9 ± 0.1	861 ± 37	8.57 × 10^7^ ± 0.37 × 10^7^		99 ± 4
50 nm AuNPs (low sensitivity)^b^	0.0	33.9	48.9 ± 0.1	1119 ± 40	5.49 × 10^7^ ± 0.20 × 10^7^	63 ± 2	
1:2 dilution	0.0	33.9	48.6 ± 0.4	541 ± 35	5.39 × 10^7^ ± 0.35 × 10^7^	63 ± 4	98 ± 6

^a^Sensitivity reduced to 2%

^b^Sensitivity reduced to 3%

### Application to the analysis of antimicrobial nanomaterials

**Fig. 5:**
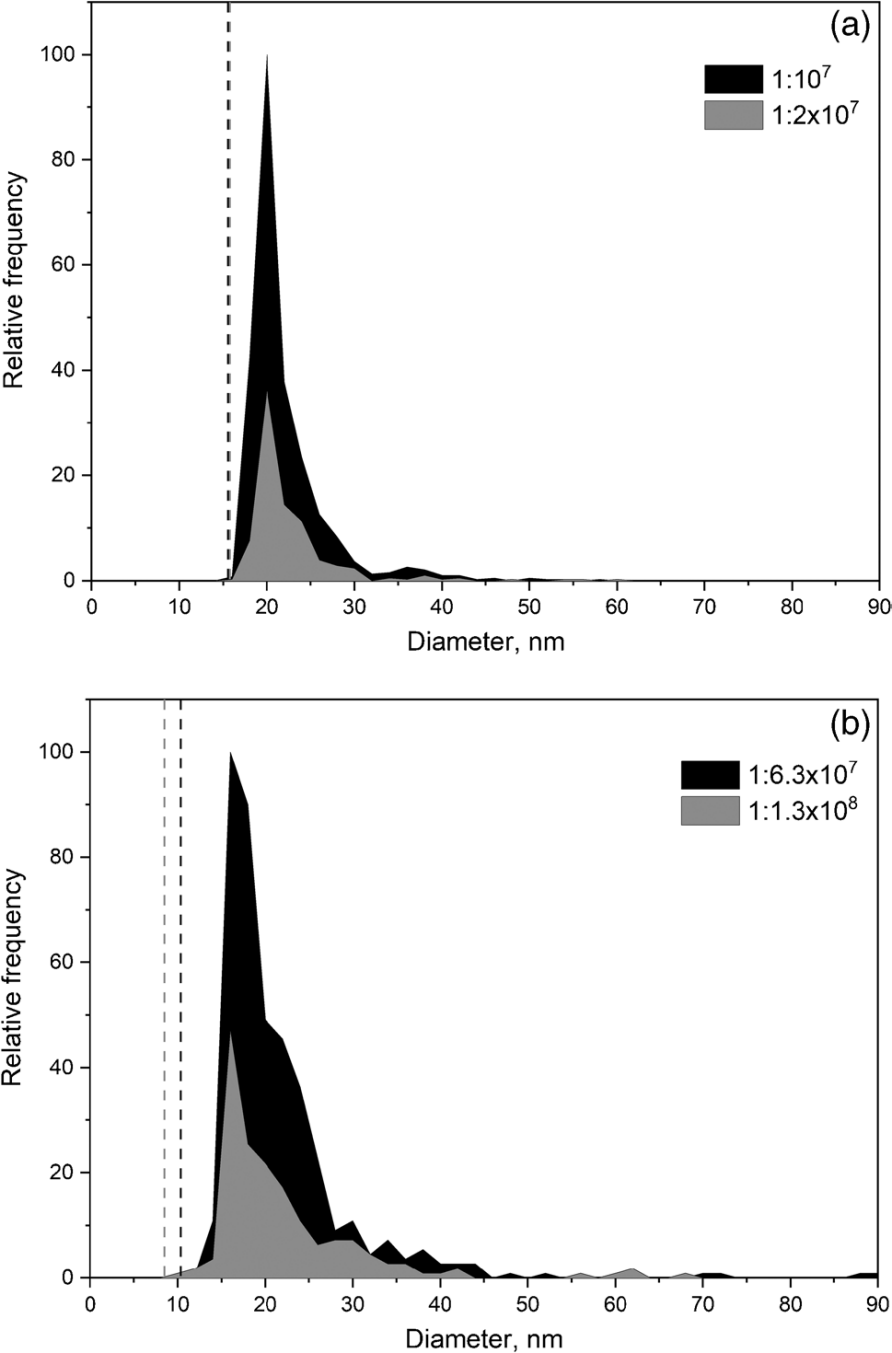
Size distributions of antimicrobial nanomaterials (**a**) M1 at 1:10^7^ (black) and 1:2 × 10^7^ (grey) dilutions; (**b**) M2 at 1:6.3 × 10^7^ (black) and 1:1.3 × 10^8^ (grey) dilution. Dwell time: 100 µs. Dashed lines: size critical values at the corresponding dilution

Two antimicrobial nanomaterials (M1 and M2), presented as aqueous suspensions containing silver nanoparticles, were subjected to analysis by SP-ICP-MS. The original suspensions had a silver content of 143 ± 4 and 736 ± 25 mg L^−1^, respectively, determined by atomic absorption spectrometry (AAS). Additional preliminary analyses showed that the suspensions contained silver nanoparticles, although also had a significant content of dissolved silver. Both suspensions were dilute down to around 1:10^7^ to reduce the contribution of the dissolved silver to blank levels, following the procedure described in Fig. 2. In the case of the analysis of M1, the total acquisition time had to be increased up to 5 min to record a significant number of particle events. Figure 5 shows the size distributions obtained for both nanomaterials for two succesive dilutions. The recorded distributions were in the range of ca. 20–60 nm, with mean diameters of 20–25 nm (Table 5). The size critical values calculated from the measured baselines were in the range of 8–15 nm, corresponding to the lower ends of the recorded distributions and hence indicating that part of the original size distributions would had been omitted. In addition, the differences observed in the number concentrations obtained for the two successive dilutions of the suspensions shown in Table 5 also suggest the partial recording of the original distributions.

**Table 5:** Mean size and number/mass concentration information of antimicrobial nanomaterials analyzed by SP-ICP-MS, HDC-ICP-MS, AAS, FESEM, and TEM. Mean ± standard deviation (*n* = 3 replicates for SP-ICP-MS, HDC-ICP-MS, and AAS; *n* = ca. 400 particles for FESEM and TEM)

Nanomaterial		SP-ICP-MS						HDC-ICP-MS			AAS	FESEM	TEM
								Ag NPs		Ag(I)	Total Ag		
	Dilution	Acquisition time s	Mean baseline intensity counts	$\mathrm X_{\mathrm C}^{\mathrm{size}}$ nm	Mean size nm	Number of events	Number concentration L^−1^	Mean size nm	Mass concentration mg L^−1^	Mass concentration mg L^−1^	Mass concentration mg L^−1^	Mean size nm	Mean size nm
M1	1:10^7^	300	0.13	15.3	19.6 ± 0.9	917 ± 40	8.2 × 10^13^ ± 0.4 × 10^13^	10 ± 2	43 ± 5	104 ± 5	143 ± 4	17 ± 9	9 ± 3
	1:2 × 10^7^	300	0.09	15.3	20.1 ± 1.1	316 ± 25	5.6 × 10^13^ ± 0.4 × 10^13^						
M2	1:6.3 × 10^7^	60	0.07	10.3	24.1 ± 0.6	465 ± 27	1.3 × 10^15^ ± 0.1 × 10^15^	12 ± 2	213 ± 14	515 ± 46	736 ± 25	29 ± 10	15 ± 8
	1:1.3 × 10^8^	60	0.04	8.2	24.5 ± 1.0	175 ± 19	9.5 × 10^14^ ± 1.0 × 10^14^						

Both nanomaterials were also analyzed by HDC-ICP-MS and electron microscopy (FESEM and TEM) for their detailed characterization. HDC allowed the simultaneous separation and the direct quantification of nanoparticulate and dissolved forms of silver [23]. Figure S2 shows the chromatograms corresponding to both nanomaterials, showing a first peak corresponding to nanoparticles of 10–11 nm and a second one to the dissolved silver. The mass concentration of silver nanoparticles and dissolved silver determined by HDC-ICP-MS is summarized in Table 5, accounting for 29 and 71%, respectively, in both samples. On the other hand, the total silver quantified by HDC-ICP-MS was in agreement with the total contents measured by AAS (recoveries of 97 and 101% in M1 and M2, respectively). Electron microscopy was used to visualize the nanoparticles in the samples to obtain information concerning their size and shape. Figures S3 and S4 show the FESEM and TEM images obtained from the samples, as well as the size distributions obtained by measuring around 400 particles per sample. The mean sizes and the standard deviations of the size distributions are summarized in Table 5. The apparent bias beetwen TEM and FESEM results can be justified by the fact that the smallest particles were not visible in FESEM, because their sizes were below the resolution limit and led to measure only the bigger particles, biasing the average size to higher diameters; furthermore, TEM image selection for observations tends to benefit the areas with small particles.

The results summarized in Table 5 show that both nanomaterial suspensions contained silver nanoparticles of ca. 10–15 nm together with dissolved silver in higher proportions, making difficult the detection and characterization of the nanoparticles by SP-ICP-MS. In fact, the size critical values achieved after dilution were similar to the mean sizes of the nanoparticles, which justifies the overestimation of the sizes measured by SP-ICP-MS. On the other hand, the affirmation that in view of the distributions shown in Fig. 5, smaller nanoparticles have not been detected is totally justified, confirming the validity of the approach proposed.

## Conclusions

Unlike conventional analytes, whose detection only requires to be at concentrations above their (concentration) limit of detection, nanoparticles must be larger than the size critical value. As a consequence, and because nanoparticles occur as polydisperse size distributions, if the whole population of particles is not detected, the quantitative information derived from these measurements will overestimate the mean size of the nanoparticles and underestimate their number concentration. In such circumstances, the validity of the “quantitative” information provided is questionable and only qualitative/semiquantitative information should be reported in the form of “the sample contains (nano)particles bearing certain element” or “the sample contains (nano)particles over a certain size and number concentration bearing certain element.” The proposed approach, based on successive dilutions and estimation of the size critical values in a systematic way, adds a quality control procedure to confirm the quantitative information if the size distribution recorded is over the size critical value estimated in the sample under study, otherwise the information should be considered only qualitative or semiquantitative.

## Supplementary Information

Below is the link to the electronic supplementary material.

Supplementary file1 (PDF 1018 KB)

Supplementary file2 (XLSX 21 KB)
