# Supplementary material for: How to trust size distributions obtained by single particle inductively coupled plasma mass spectrometry analysis
Source: Anal Bioanal Chem. 2022 Jul 30;415(11):2101–12. doi: 10.1007/s00216-022-04215-z (PMC10079697; doi:10.1007/s00216-022-04215-z)

HOW TO TRUST SIZE DISTRIBUTIONS OBTAINED BY SINGLE PARTICLE INDUCTIVELY PLASMA MASS SPECTROMETRY ANALYSIS

Ana C. Gimenez-Ingalaturre, Khaoula Ben Jeddou, Josefina Pérez-Arantegui, María S. Jiménez, Eduardo Bolea, Francisco Laborda

Group of Analytical Spectroscopy and Sensors (GEAS)
Institute of Environmental Sciences (IUCA)
University of Zaragoza
Pedro Cerbuna 12, 50009 Zaragoza, Spain.

Supplementary material

Instrumental and data acquisition parameters

Table S1. Instrumental and data acquisition parameters for HDC-ICP-MS.

ICP-MS parameters	
RF power	1200 W
Argon flow rate	
Plasma	
Auxiliary	1.2 L min^{-1}
Nebulizer	0.8 L min^{-1}
**Data acquisition parameters**	
Dwell time	50 ms
Sweeps	1
Isotopes monitored	^{107}Ag, ^{109}Ag

HDC parameters	
Column type	PL-PSDA type 1, 5-300 nm
Mobile phase	0.34 mM SDS + 1mM PA
Flow rate	1.6 mL min^{-1}
Injection volume	50 µL

SP-ICP-MS data processing by using SPCal software

Raw data of the measurements of 40 nm silver nanoparticles spiked with increasing concentrations of ionic silver were processed using the open-source software SPCal version 0.7.1. The raw data was processed by using the Poisson filter, the epsilon parameter value was set to 0, because no significant effect was observed when varied from 0 to 1. Results obtained are presented in table S3 and size distributions are presented in figure S1.

Table S2. Mean size and number concentration of nanoparticles, size critical values (X_C^{size}) and nanoparticle recovery for 40 nm silver nanoparticles spiked with increasing concentrations of ionic silver for dwell time of 100 µs. Total acquisition time: 60 s. Mean ± standard deviation (n=3).

suspension	mean baseline intensity counts	X_C^{size} nm	mean size nm	number concentration L^{-1}	recovery %
40 nm AgNPs	0.06	20.6	38.1 ± 0.2	$1.51x10^8 \pm 0.03x10^8$	101 ± 2
40 nm AgNPs + 0.02 $\mu g\ L^{-1}$ Ag(I)	0.1	20.8	38.6 ± 0.2	$1.36x10^8 \pm 0.01x10^8$	96 ± 1
40 nm AgNPs + 0.15 $\mu g\ L^{-1}$ Ag(I)	0.5	22.7	38.3 ± 0.2	$1.29x10^8 \pm 0.01x10^8$	89 ± 1
40 nm AgNPs + 0.26 $\mu g\ L^{-1}$ Ag(I)	1	24.5	39.7 ± 0.1	$1.19x10^8 \pm 0.03x10^8$	82 ± 2
40 nm AgNPs + 1.34 $\mu g\ L^{-1}$ Ag(I)	5	31.4	43.8 ± 0.1	$7.9x10^7 \pm 0.4x10^7$	55 ± 3
40 nm AgNPs + 2.44 $\mu g\ L^{-1}$ Ag(I)	11	36.0	47.6 ± 0.1	$4.4x10^7 \pm 0.1x10^7$	30 ± 1

Fig. S1. Size distributions of 40 nm silver nanoparticles spiked with increasing concentrations of ionic silver for dwell times of 100 µs. The legends list the ionic silver concentration in the nanoparticle suspensions.

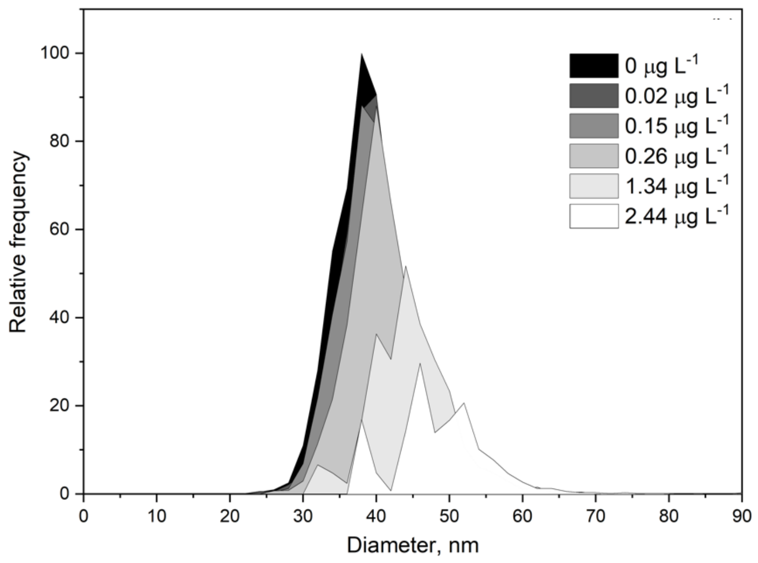

Analysis of antimicrobial nanomaterials by HDC-ICP-MS

Figure S2. Hydrodynamic chromatograms of antimicrobial materials (a) M1 and (b) M2. First peak: Ag nanoparticles, second peak: dissolved Ag.

(a)

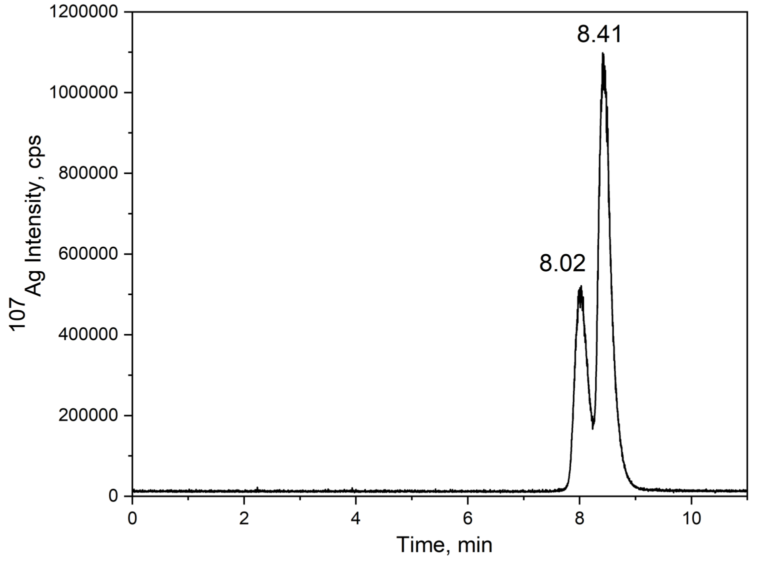

(b)

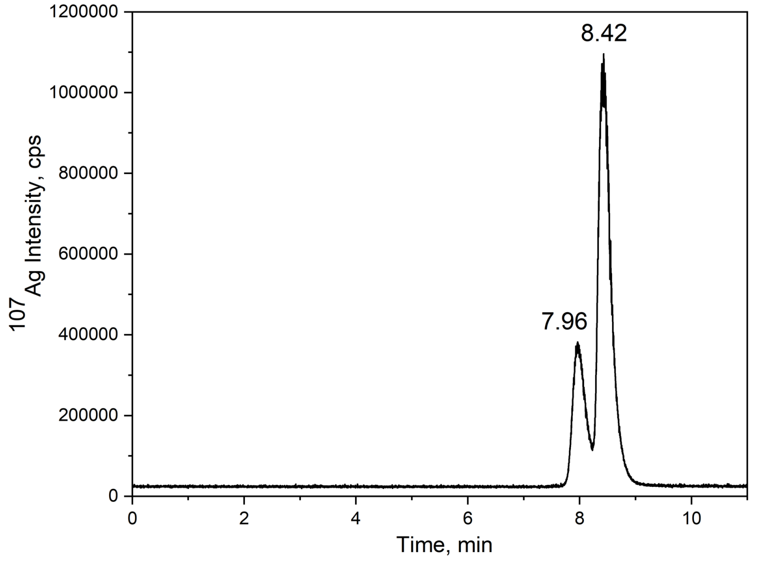

Analysis of antimicrobial nanomaterials by FESEM and TEM

Figure S3. FESEM micrographs and size distributions of antimicrobial nanomaterials (a) M1 and (b) M2.

(a)

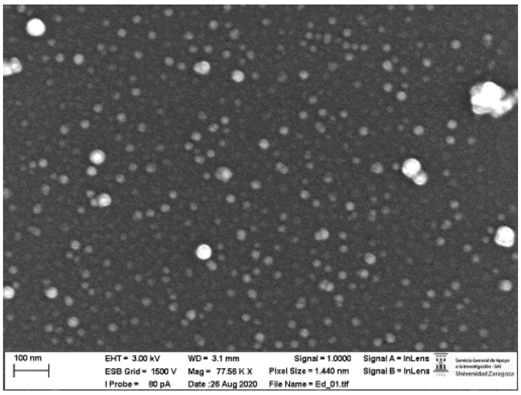

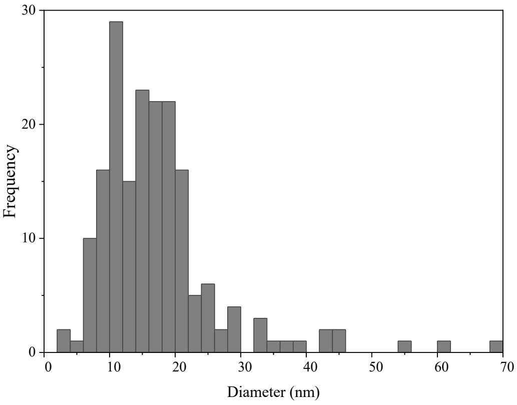

(b)

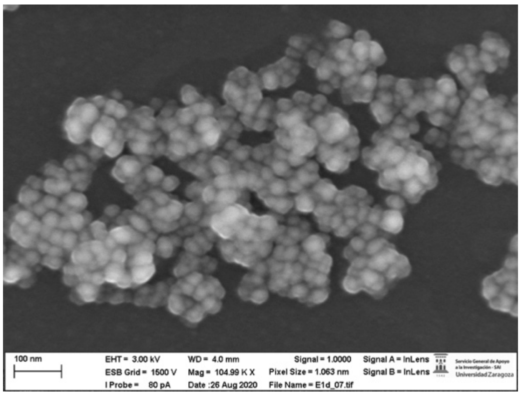

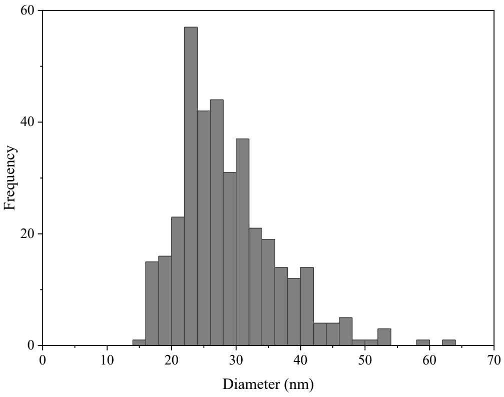

Figure S4. TEM micrographs and size distributions of antimicrobial nanomaterials (a) M1 and (b) M2.

(a)

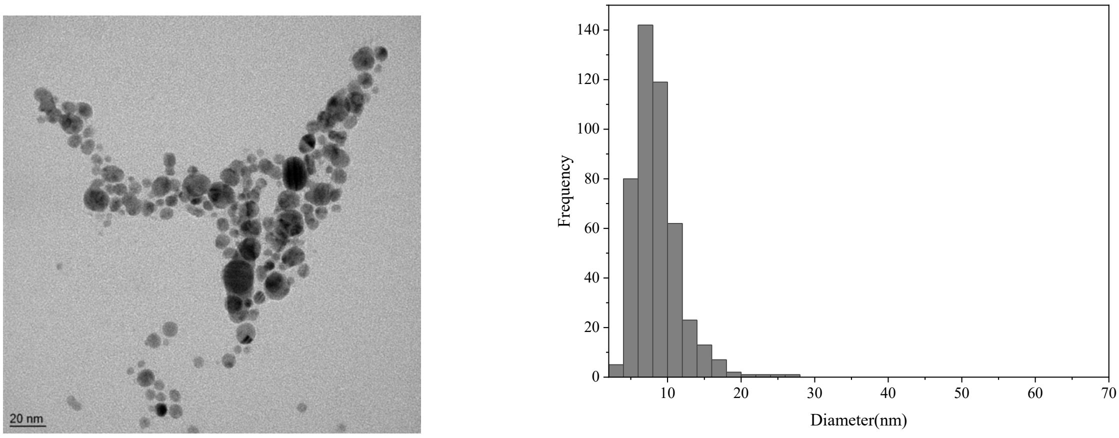

(b)

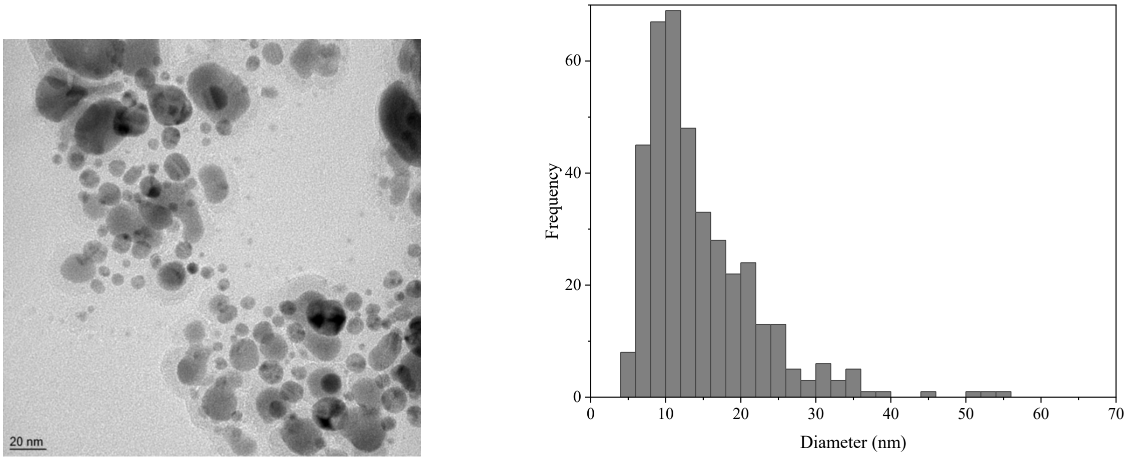

Supplement: Supplementary file 1 — Supplementary file1 (PDF 1018 KB) [file 216_2022_4215_MOESM1_ESM.pdf]
